# Hot topics in psychiatry/psychopharmacology

**DOI:** 10.1192/j.eurpsy.2025.73

**Published:** 2025-08-26

**Authors:** P. Mohr

**Affiliations:** 1 National Institute of Mental Health - CZ, Klecany; 2 3rd Faculty of Medicine, Charles University, Prague, Czech Republic

## Abstract

**Abstract:**

Psychiatry faces numerous challenges that significantly affect mental health of people in Europe, from climate change, wars, migration, pandemics, to rapid advance of artificial intelligence. Among the most pressing issues are deteriorating mental health of children and adolescents and the lack of trained professionals. At the same time, progress in neuroscientific research contributes to the development of new effective treatments, e.g., monoclonal antibodies for Alzheimer’s disease; repurposing of drugs for psychiatric disorders, such as GLP-1 agonists, showed potential in treatment of alcohol addiction. There is also a renewed interest in psychedelics, recent studies verified their therapeutic efficacy in treatment of several mental disorders. Neurostimulation methods (rTMS, tDSC) are recognized non-pharmacological interventions; utilization of new technologies (AI, virtual reality, mobile apps, etc.) can help us in treatment and prevention of mental disorders.

**Disclosure of Interest:**

None Declared

